# Draft Genome Sequence of Leishmania tarentolae Parrot Tar II, Obtained by Single-Molecule Real-Time Sequencing

**DOI:** 10.1128/MRA.00050-20

**Published:** 2020-05-21

**Authors:** Yasuyuki Goto, Akihiro Kuroki, Kengo Suzuki, Junya Yamagishi

**Affiliations:** a Laboratory of Molecular Immunology, Department of Animal Resource Sciences, Graduate School of Agricultural and Life Sciences, The University of Tokyo, Tokyo, Japan; b euglena Co., Ltd., Tokyo, Japan; c Microalgae Production Control Technology Laboratory, RIKEN, Yokohama, Japan; d Research Center for Zoonosis Control, Hokkaido University, Sapporo, Japan; e Global Station for Zoonosis Control, GI-CoRE, Hokkaido University, Sapporo, Japan; Broad Institute

## Abstract

Leishmania tarentolae is a protozoan parasite of lizards and is nonpathogenic to mammals. Genome information for the nonpathogenic species will facilitate an understanding of the pathology caused by species pathogenic to mammals. Here, we report resequencing of the L. tarentolae genome with a single-molecule real-time (SMRT) sequencer to provide a more complete genome assembly.

## ANNOUNCEMENT

Protozoan parasites of the genus *Leishmania* are causative agents of leishmaniasis. There are around 20 species causing the disease in humans, which can be mainly classified into three forms, namely, cutaneous leishmaniasis, mucocutaneous leishmaniasis, and visceral leishmaniasis, depending on the infecting species. Genetic comparison of *Leishmania* species causing different disease forms has facilitated understanding of the pathology and immunology of the disease (1). In addition, the genetic information is useful for identification of leishmanial targets good for the development of drugs, vaccines, or diagnostic tests. For example, we previously reported that antigens of serological significance can be identified *in silico* by searching the parasite genome for genes encoding proteins with tandem repeats (TRs) (2). To identify molecules responsible for the virulence of *Leishmania* parasites, genetic comparison between pathogenic and nonpathogenic species is a useful approach. This is why Leishmania tarentolae, which is found in lizards and is thought to be nonpathogenic to mammals, was sequenced, and its genome was compared with that of pathogenic *Leishmania* species (3). The L. tarentolae genome lacks many of the TR genes found in other *Leishmania* parasites, which represents either evolutionary loss of genes responsible for parasitism to mammals or just annotation errors due to short-read sequencing using Roche 454 and Illumina systems.

In order to elucidate the loss of TR genes in L. tarentolae, we resequenced the genome of L. tarentolae Parrot Tar II (ATCC 30267). Total DNA was isolated, using the blood and cell culture DNA midikit (Qiagen), from promastigotes grown in brain heart infusion medium supplemented with 10% heat-inactivated fetal bovine serum and 10 μg/ml hemin. Five micrograms of DNA was fragmented to 20 kbp using a g-TUBE (Covaris, Inc.) and ligated with adaptors (SMRTBell template preparation kit v1.0) to prepare the SMRTBell structure. The resulting library was sequenced with a PacBio RS II system (Pacific Biosciences, USA) using 4 single-molecule real-time (SMRT) cells.

Using the HGAP3 assembler (with default parameters except as follows: expected genome size, 30 Gbp; target coverage, 30), a polished sequence with 510,681 reads covering 4,313,522,161 nucleotides and an assembled sequence with 35,416,496 bp in 179 contigs (genome coverage, ∼120×; maximum length, 2,700,252 bp; *N*_50_, 663,019 bp; GC content, 57.41%) were obtained. The genome was larger than that of L. tarentolae in the previous paper (30,440,719 bp) (3), Leishmania major (32,816,678 bp), Leishmania infantum (32,134,935 bp), and Leishmania braziliensis (32,005,207 bp) (1, 4).

Comparison between the previous and current L. tarentolae genomes revealed that the size difference (∼5 million bp) can be partly explained by missing repeat sequences in the previous version. For example, a gene containing multiple copies of a 219-bp repeat unit can be found in all three pathogenic *Leishmania* species but is absent in the previous L. tarentolae genome; our data show that the repeat motif is also present in L. tarentolae, although it has a nonsense mutation within the repeat domain. Thus, resequencing of parasite genomes with a long-read sequencer can contribute to accurate identification of repeat motifs in the genomes, which may lead to an understanding of the evolution of TR genes and their significance for adaptation to mammals.

### Data availability.

These data are available under GenBank assembly accession number GCA_009731335 and SRA accession number DRX197845.
